# Manual action re-planning interferes with the maintenance process of working memory: an ERP investigation

**DOI:** 10.1007/s00426-022-01741-4

**Published:** 2022-11-24

**Authors:** Rumeysa Gunduz Can, Thomas Schack, Dirk Koester

**Affiliations:** 1 Faculty of Psychology and Sports Science, Department of Sports Science, Neurocognition and Action-Biomechanics Research Group, Bielefeld University, Bielefeld, Germany; 2 Institute of Sport and Exercise Sciences, Department of Sport and Exercise Psychology, University of Münster, Münster, Germany; 3 Center for Cognitive Interaction Technology (CITEC), Bielefeld University, Bielefeld, Germany; 4 Faculty of Business and Management, Department of Business Psychology, Business School Berlin, Berlin, Germany

## Abstract

The current study investigated the re-planning of the grasping movements, its functional interactions with working memory (WM), and underlying neurophysiological activity. Mainly, the current study investigated the movement re-planning interference with WM domains (verbal, visuospatial) and processes (maintenance, retrieval). We combined a cognitive-motor dual-task paradigm with an EEG setting. Thirty-six participants completed the verbal and visuospatial versions of a WM task concurrently with a manual task which required performing a grasp-and-place movement by keeping the initial movement plan (prepared movement condition) or changing it for reversing the movement direction (re-planned movement condition). ERPs were extracted for the prepared and re-planned conditions in the verbal and visuospatial tasks separately during the maintenance and retrieval processes. ERP analyses showed that during the maintenance process of both the verbal and visuospatial tasks, the re-planned movements compared to the prepared movements generated a larger positive slow wave with a centroparietal maximum between 200 and 700. We interpreted this ERP effect as a P300 component for the re-planned movements. There was no ERP difference between the planned and re-planned movements during the retrieval process. Accordingly, we suggest that re-planning the grasp-and-place movement interfered at least with the maintenance of the verbal and visuospatial domains, resulting in the re-planning costs. More generally, the current study provides the initial neurophysiological investigations of the movement re-planning–WM interactions during grasping movements, and contributes to a better understanding of the neurocognitive mechanisms underlying manual action flexibility.

**Supplementary Information:**

The online version contains supplementary material available at 10.1007/s00426-022-01741-4.

## Introduction

Humans plan and execute goal-directed motor actions by incorporating various physiological and cognitive factors such as muscle fatigue or action intentions, with the environmental factors such as target object location (for reviews, see Glover, 2004; Hommel et al., 2016). Those factors often change continuously and sometimes even unexpectedly in the dynamic physical world. Therefore, satisfying the desired action outcomes requires not only the thoughtful planning and execution but also the fast and smooth adaptations to changing action demands (e.g., Morsella, 2009). Accordingly, action flexibility is an essential cognitive ability that enables a rich repertoire of skilled motor actions by organizing, monitoring and adapting actions with minimal time and effort (e.g., Gentsch et al., 2016; Verbruggen et al., 2014). One way to achieve action flexibility during ongoing motor actions is movement re-planning, which includes changing a prepared movement plan favoring an alternative plan (e.g., Pouget et al., 2017; Ullsperger et al., 2014a, b).

Manual actions, which humans frequently perform to achieve daily life routines, communicate with others, do sports, or play musical instruments, constitute a significant share of the motor action repertoire. Mainly, grasping movements are the most frequently performed, yet the most complex manual actions requiring the close engagement of the sensorimotor systems and cognitive processes (e.g., Castiello, 2005; Grafton, 2010). The current study investigated the re-planning of grasping movements, its functional interactions with working memory (WM) and underlying neurophysiological activity. With this, we aimed for contributing to a better understanding of the neurocognitive mechanisms underlying manual action flexibility, i.e., how the human brain orchestrates the sensorimotor systems with cognitive processes to plan, execute and adapt manual actions in the dynamic physical world.

During movement re-planning, changing the prepared movement plan requires the cancelation of an inappropriate initial plan, and the selection and preparation of an appropriate new plan (e.g., Georgopoulos et al., 1981; Soechting & Lacquaniti, 1983). Accordingly, movement re-planning is accompanied by additional cognitive operations that are not required during movement planning and execution. For example, a change in target object location during grasping movements reverses the movement direction, thus changing the desired action outcome. In that situation, satisfying the new action outcome requires changing the initial plan prepared based on the original movement direction (e.g., Quinn & Sherwood, 1983; for a review, see Elliott et al., 2017). Accordingly, error detection mechanisms evaluate whether the initial plan still satisfies the desired action outcome (e.g., Aron et al., 2014). If necessary, motor inhibition mechanisms suppress the movement planned in the original direction (e.g., Hartwigsen & Siebner, 2015). Moreover, a new movement plan is selected and prepared based on the reversed movement direction (e.g., Hartwigsen et al., 2012). These operations are accompanied by decision-making and conflict resolution mechanisms, for example, for balancing the execution of the planned movement and the need for overriding it based on the new action outcome (e.g., Steinhauser & Yeung, 2010). Furthermore, all these operations also need to be well-monitored for a prompt cancelation of the initial plan, yet with an adequate time for the efficient selection and preparation of the new plan. Accordingly, movement re-planning is an intentional and cognitively demanding process.

Previous research has suggested that humans achieve successful movement re-planning but at the expense of the motor and cognitive costs (e.g., Pouget et al., 2017; Ullsperger et al., 2014a, b). Such costs have been also reported for manual actions (e.g., Logan & Fischman, 2011, 2015; Spiegel et al., 2012, 2013, 2014; Weigelt et al., 2009). For example, Spiegel and colleagues (2013) examined the cognitive costs of the movement re-planning by focusing on the functional interactions of the grasping movements with WM. In a cognitive-motor dual-task paradigm, participants completed a WM task (verbal and visuospatial versions) concurrently with a manual task. The manual task required performing a grasp-and-place movement by keeping the initial movement plan (prepared movement condition) or changing it (re-planned movement condition). Comparisons of the memory performance for the verbal and visuospatial tasks between two movement conditions revealed the lower memory performance for both tasks in the re-planned condition than the prepared condition (Spiegel et al., 2013). The authors interpreted the lower memory performance indicating that additional cognitive operations involved in the movement re-planning interfered with the verbal and visuospatial domains and decreased the memory performance for both WM tasks. Moreover, the authors proposed that the re-planning interference originated from the domain-general cognitive resources shared between the movement re-planning and both WM domains, indicating the functional interactions of the movement re-planning with both the verbal and visuospatial domains.

In line with the cognitive costs, it has also been shown that during grasping movements, a variety of the strategies are implemented for eliminating those costs, such as recalling the previous grasp postures (e.g., Hughes et al., 2012; Hughes & Seegelke, 2013) or hand paths (e.g., Jax & Rosenbaum, 2007; Van der Wel et al., 2007). Additionally, movement re-planning–WM interactions during grasping movements are also compatible with the motor control research suggesting the functional role of WM in manual actions. For example, it has been shown that WM selects, prepares and changes the movement plans (e.g., Fournier et al., 2014; Gallivan et al., 2016) as well as keeps the task-related target information active for the upcoming movement (e.g., Hesse & Franz, 2009, 2010; Hesse et al., 2016; Kohler et al., 1989; for a review, see Schenk & Hesse, 2018).

Previous research has suggested that movement re-planning is critical for satisfying the desired action outcomes during ongoing motor actions. Previous research has also suggested that grasping movements interact with WM not only during the movement planning and execution but also during the movement re-planning. However, only limited behavioral research has systematically investigated the functional interactions of the movement re-planning with WM during grasping movements (e.g., Logan & Fischman, 2011, 2015; Spiegel et al., 2012, 2013, 2014; Weigelt et al., 2009). Moreover, there has been no research systematically investigating the cortical mechanisms underlying those interactions to the best of our knowledge. Accordingly, we aimed for bridging this research gap and contributing further to the understanding of the neurocognitive mechanisms underlying manual action flexibility. The current study investigated the neurocognitive mechanisms by focusing on the neurophysiological correlations of the movement re-planning–WM interactions. That is, how and to what extent would changing the initial plan of a grasping movement interact with WM. More importantly, what the neurophysiological correlates of this interaction would be.

We adapted a well-established cognitive-motor dual-task paradigm from the previous behavioral studies to the electroencephalography (EEG) setting (e.g., Spiegel et al., 2012, 2013, 2014). We also demonstrated this paradigm's EEG adaptability in our previous ERP study, which investigated the neurophysiological correlates of the movement execution–WM interactions during grasping movements (Gunduz Can et al., 2017). By comparing a baseline single-task condition with a dual-task condition, the previous study showed that mere movement execution (without movement re-planning) interfered with the visuospatial but not with the verbal domain during the encoding process. These findings were in line with the behavioral findings by Spiegel and colleagues (2013) and further provided the evidence for the domain- and process-specific interactions of the movement execution with WM.

The current dual-task paradigm included completing a WM task (verbal and visuospatial versions) concurrently with a manual task. The manual task required performing a grasp-and-place movement by keeping the initial movement plan (prepared movement condition) or changing it for reversing the movement direction (re-planned movement condition). EEG activity was recorded while participants were actively performing the movement. From the EEG recordings, event-related potentials (ERPs) were extracted. In the current study, ERPs are particularly suitable for investigating the movement re-planning–WM interactions not only for the separate WM domains but also for the separate WM processes (encoding, maintenance, retrieval, e.g., Jonides et al., 2008). Accordingly, we could examine the source of the movement re-planning–WM interactions and the underlying neurophysiological activity.

Based on the previous research, we expected that movement re-planning would entail cognitive costs for WM, referred to as the movement re-planning costs. We defined the re-planning costs as the difference between the memory performances in the prepared and re-planned movement conditions regarding the behavioral data. Based on the behavioral findings by Spiegel and colleagues (2013), we expected that movement re-planning would interfere with memorizing the verbal and visuospatial information to a similar degree, thus decreasing the memory performance for the verbal and visuospatial tasks. Accordingly, we expected that memory performance would be lower in the re-planned condition than the prepared condition independent of the WM task, indicating the behavioral domain-general re-planning costs.

We defined the re-planning costs as the difference between the ERPs in the prepared and re-planned movement conditions regarding the EEG data. Following the behavioral hypothesis, we expected that movement re-planning would interfere with the verbal and visuospatial domains at the neurophysiological level, resulting in the neurophysiological domain-general re-planning costs. Specifically, we expected ERPs in the verbal and visuospatial tasks to differ between the prepared and re-planned conditions. Previous ERP research has suggested that corrective adaptations of the ongoing motor actions generate a P300 ERP component^1^ when there is a mismatch between the initial movement plan and the desired action outcome (e.g., Chase et al., 2011; Fleming et al., 2009; Krämer et al., 2011; Randall & Smith, 2011; Trewartha et al., 2013; for the role of other factors such as feedback effects of action outcomes Holroyd & Coles, 2002; Ullsperger et al., 2014a, b). We expected the re-planned movements to generate a larger P300 than the prepared movements independent of the WM task based on these findings.

## Methods

### Participants

Thirty-six right-handed participants (17 females, *M* age = 23.64 years, SD = 2.95) from the students of Bielefeld University participated in the study. All participants had the normal or correct-to-normal vision and no known neurological disorder.

After the outlier exclusion, we included 34 participants (15 females, *M* age = 23.53 years, SD = 2.94) in the statistical analysis of the memory performance. There was no outlier exclusion for the movement execution time. After the EEG data pre-processing and outlier exclusion, for the maintenance process, we included 26 participants (14 females, *M* age = 24.04 years, SD = 3.01) and 26 participants (15 females, *M* age = 23.88 years, SD = 2.96) for the first and second ERP analyses. For the retrieval process, it was 26 participants (13 females, *M* age = 23.50 years, SD = 3.13) and 25 participants (12 females, *M* age = 23.52 years, SD = 3.19) for the first and second ERP analyses.

Participants provided written informed consent and received either 15-Euro or 2-h participation credits as compensation. We conducted the study confirming the ethical standards of the sixth revision of the Declaration of Helsinki and the approval of the ethics committee of Bielefeld University.

### Materials

**Fig. 1:**
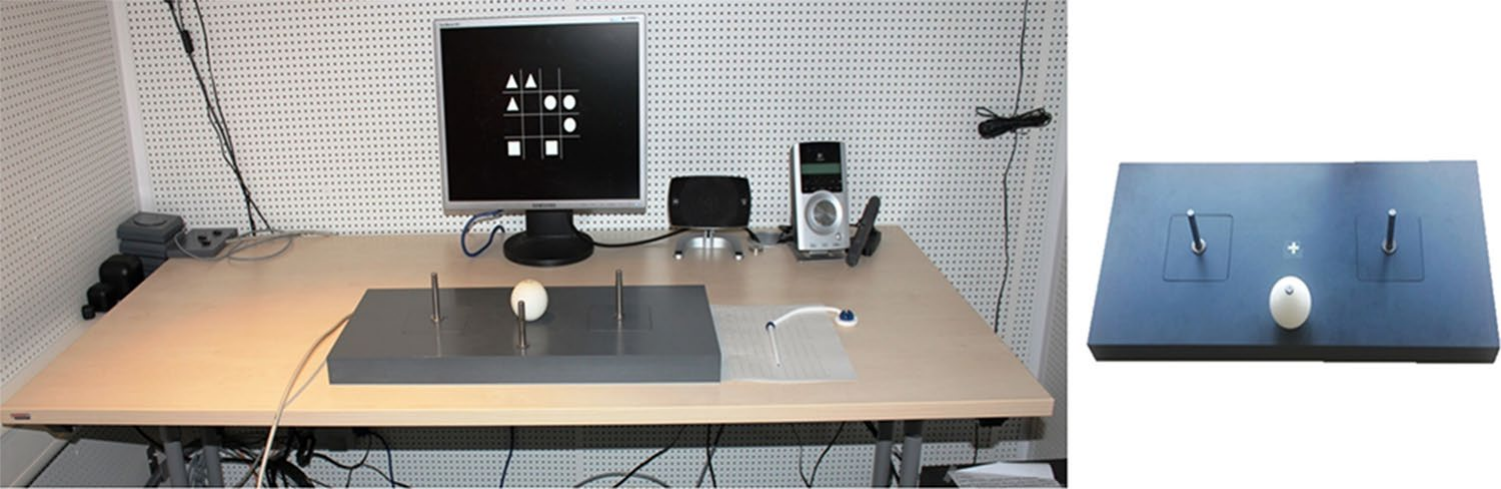
Experimental set-up (left) and task board (right). In the figure, the experimental set-up shows the example stimulus and answer sheet for the visuospatial task. The task board consisted of three sticks as the start position and two motor targets (left and right). A yellow cross marked the center of the task board. A sphere, placed on the start position as shown in the figure, was placed on one of the motor targets

We presented the stimulus event for the experimental task on a 17-in flat-screen monitor with integrated speakers and a resolution of 1024 × 768 pixels (see Fig. 1).

WM task had two versions, verbal task and visuospatial task. Stimuli for the verbal task were one hundred letter sequences consisting of eight consonants of the Latin alphabet. Each consonant was 2 cm in height and width. There was neither any abbreviation nor alphabetic order among the consonants. Each letter sequence was along a vertical axis at the center of the monitor screen, thus avoiding any possible visual field effect. Stimuli for the visuospatial task were one hundred 4 × 4 matrices consisting of sixteen equiprobable positions. There was a variation of the eight symbols within each matrix selected from three categories, i.e., triangle, circle, square, and placed at any random position. Each symbol was 2 cm in height and width (see Fig. 1 for an example stimulus for the visuospatial task).

The manual task required grasping a sphere, holding it, and placing it on a motor target, i.e., grasp-and-place movement. We used a task board (4 × 60 × 28 cm) consisting of three sticks (10 cm in height, 0.5 cm in width) and a yellow cross. The yellow cross marked the center of the board. One stick served as a start position. The other two sticks, mounted on the board being 15 cm away from the yellow cross, served as the left and right motor targets (see Fig. 1). Participants placed the sphere, 6 cm in diameter and furnished with a hole of 10 mm, on one of the motor targets. We equipped the start position and motor targets with pressure-sensitive micro switches, providing participants with the self-paced trial starts and ends.

We used two auditory sinusoidal tones of 400 Hz (low tone) and 750 Hz (high tone) as a keep/change cue for movement planning.

### Procedure and design

After giving written informed consent, participants sat comfortably in an electrically shielded cabin where the study took place.

**Fig. 2:**
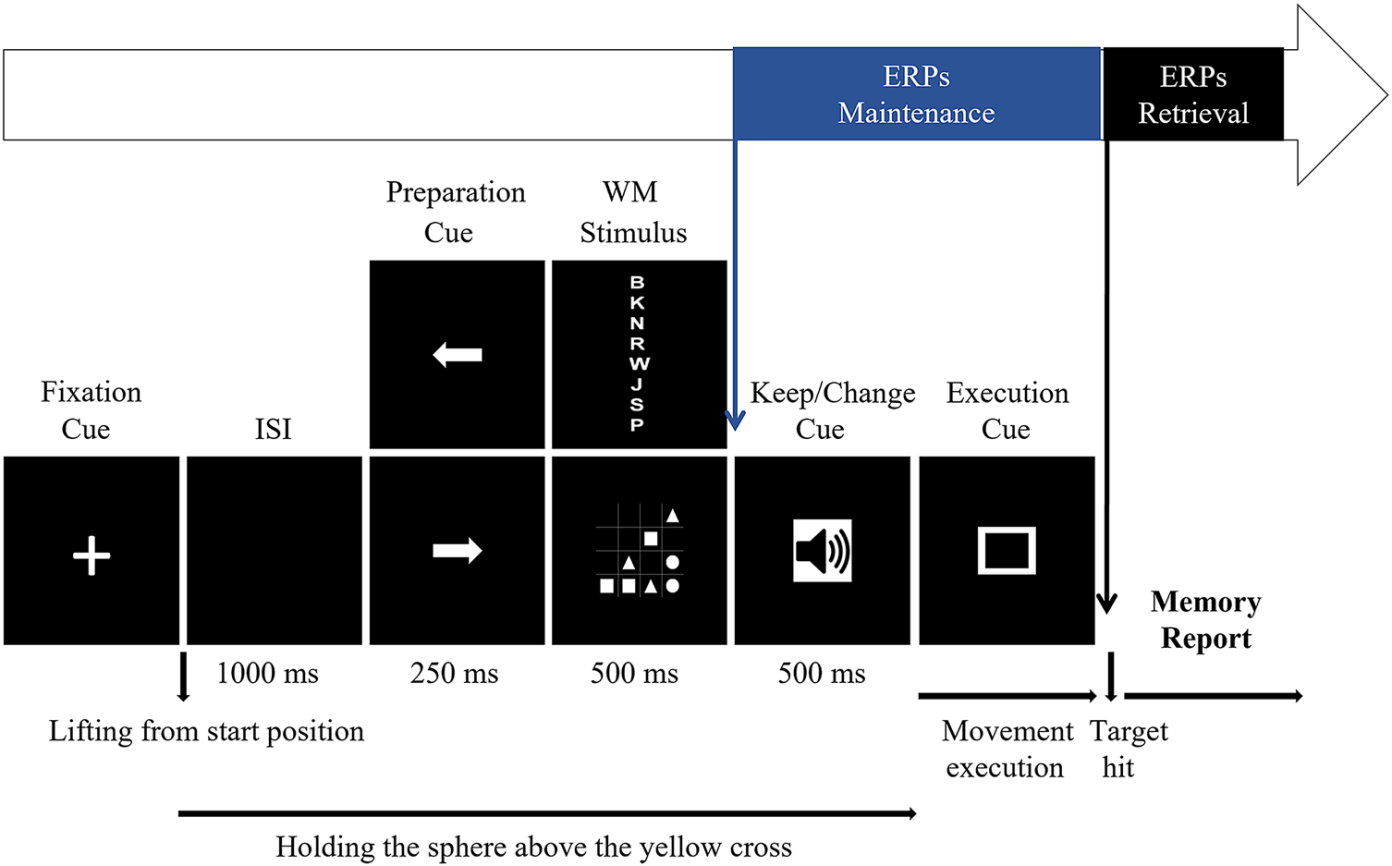
Timing of the stimulus event for a trial, including the reference events for the ERPs related to the maintenance and retrieval processes. The maintenance epochs were time-locked to the keep/change cue onset. The retrieval epochs were time-locked to the target hit

The experimental task required completing a WM task concurrently with a manual task. Participants themselves started and ended the fixed sequence of the stimulus event (see Fig. 2). In the beginning, the sphere was at the start position. The stimulus event started when participants grasped the sphere, pressed it down on the start position and subsequently lifted it from there. Lifting the sphere from the start position opened a 1000 ms inter-stimulus interval (ISI). During ISI, participants transported the sphere to the yellow cross and held it there until an upcoming movement execution cue. While participants were holding the sphere, they first received a movement preparation cue as an arrow (250 ms duration) pointing towards one of the left or right motor targets, thus indicating the movement direction. We considered that participants initially planned the movement based on the preparation cue. First, until an upcoming keep/change cue, participants did not know whether they would execute the movement in the original or reversed movement direction. Second, participants executed the movement in the original direction for most trials. Therefore, it was more efficient to plan the movement based on the preparation cue. In line with this consideration, Spiegel and colleagues (2012) showed that participants tended to hold the sphere closer to the pointed motor target rather than holding it directly above the yellow cross. The authors interpreted this tendency indicating that participants initially planned the movement based on the pointing direction of the arrow (Spiegel et al., 2012).

After the preparation cue, participants received a WM stimulus, either a letter sequence or a 4 × 4 matrix (500 ms duration). Then, they received the keep/change cue as one of the low or high auditory tones (500 ms duration). If the auditory tone served as a keep cue, participants placed the sphere on the pointed motor target, thus keeping the initial movement plan, i.e., prepared movement condition. If the auditory tone served as a change cue, participants reversed the movement direction and placed the sphere on the opposite motor target. That is, they changed the initial plan, i.e., re-planned movement condition (e.g., Quinn & Sherwood, 1983). After the keep/change cue, participants received the movement execution cue. With the execution cue onset, participants transported the sphere from the yellow cross and placed it on the pointed or opposite motor target based on the auditory tone. The sphere placement ended the grasp-and-place movement. We termed the sphere placement as ‘target hit’ regardless of whether the sphere was placed on the correct motor target. Participants started reporting the memory items immediately after the target hit. Then, they placed the sphere back on the start position for the subsequent trial.

The experimental task did not prioritize one task over the other. Participants memorized as many letters or symbols as possible and moved the sphere as quickly as possible but at a comfortable speed. We considered any trial a ‘placement error trial’ if the sphere was placed on the wrong motor target. The verbal and visuospatial tasks required a written report on the answer sheets provided. The verbal task required memorizing the letters and reporting them independently of the serial order (only the identity). The answer sheet consisted of rectangle blank boxes with a left to right orientation. The visuospatial task required memorizing the symbols and reporting the correct symbols in the correct position within the matrix (identity and position). The answer sheet consisted of 4 × 4 matrices.

The current study was based on a 2 × 2 within-subject design with the factors *WM Task* (verbal vs visuospatial) and *Movement Planning* (prepared vs re-planned). Each participant performed the experimental task in two experimental blocks, verbal task block and visuospatial task block. Each block consisted of 100 experimental trials, from which 70 trials were for the prepared and 30 trials were for the re-planned movement condition (e.g., Smeets et al., 2016). There were four experimental conditions: Prepared and re-planned movement conditions in the verbal task and prepared and re-planned movement conditions in the visuospatial task.

There were four experimental groups (each had 9 participants) based on the order of the task blocks and the keep/change cue that the auditory tone served. Two groups (A and C) completed the visuospatial task as the first block. While group A received the low tone as the keep cue (consequently the high tone as the change cue), group C received the high tone as the keep cue. The other two groups (B and D) completed the verbal task as the first block. In this case, group B and D received the low tone and the high tone as the keep cue, respectively. We randomly assigned participants to each group.

Prior to experimental blocks, participants completed 10 trials of each block for familiarization. We did not use the data from the training blocks for the statistical analyses. Between the verbal and visuospatial blocks, there was a 15-min break. Moreover, participants had self-paced breaks after the 50th trial in each block. We used Presentation software for the stimulus presentation, response registration, and timing (Neurobehavioral Systems, Albany, CA). The entire experimental session lasted about 2 h.

### EEG recording

We recorded EEG data using a 64-channel amplifier and WaveGuard EEG cap (ANT, www.ant-neuro.com) and arranged the Ag/AgCL electrodes according to the International 10–20 system (Oostenveld & Praamstra, 2001). We placed four electrodes above and below the right eye and lateral to both eyes for the ocular artifacts. Finally, we implemented the band-pass filtering of DC-138 Hz and the digitization of 512 Hz, and kept the impedance of all electrodes below 5 kV.

### Data analysis

#### Behavioral data analysis

The dependent variables were memory performance and movement execution time regarding the behavioral data. We defined memory performance as the number of correctly reported letters independently of the serial order in the letter sequence for the verbal task. It was the number of correctly reported symbols in the correct position within the matrix for the visuospatial task. We defined movement execution time as the time from the keep/change cue onset to the target hit. For the memory performance, we conducted a 2 × 2 repeated measures analysis of variance (ANOVA), including the factors *WM Task* (verbal vs visuospatial) and *Movement Planning* (prepared vs re-planned) on the arcsine transformed proportions of the correct answers. We conducted another 2 × 2 repeated-measures ANOVA for the movement execution time, including the factors *WM Task* and *Movement Planning*.

We performed the initial inspection of the behavioral data separately for the memory performance and movement execution time. First, we excluded the placement error trials from the statistical analyses. Then, we calculated the mean movement execution time for each participant and excluded the trials deviating more than three standard deviations from it. After the initial exclusion of the trials, we further inspected the data for the outliers. Based on the suggestion by Tabachnick and Fidell (2013), we used the z-score outlier criterion. Accordingly, we accepted any case as an outlier if its standardized score was larger than the z-score of 3.29 or smaller than the z-score of − 3.29. We considered that the z-score criterion depends on the standard normal distribution and sample size; hence it might be biased by the outliers. Therefore, we also examined the box plots. Then, we excluded two participants from the memory performance analysis. Nonetheless, to provide transparency for the outlier exclusion and statistical test results, we also reported the test results before the outlier exclusion (e.g., Aguinis et al., 2013; Bakker & Wicherts, 2014). Table 1 shows the percentage of the trials excluded from the statistical analyses of the memory performance and movement execution time after the initial inspection of the behavioral data.

**Table 1:** Percentage of the trials excluded from the statistical analyses of the behavioral data

Memory performance				Movement execution time			
Verbal task		Visuospatial task		Verbal task		Visuospatial task	
Prepared	Re-planned	Prepared	Re-planned	Prepared	Re-planned	Prepared	Re-planned
% 4	% 5.7	% 6.1	% 5.5	% 4	% 5.9	% 6.9	% 6.3

Memory performance trials are based on the data from thirty-four participants (two participants were excluded as the outliers from the statistical analysis). Movement execution time trials are based on the data from thirty-six participants (no participant was excluded)

We checked the normality assumption of the two-way repeated-measures ANOVAs with the Shapiro Wilks test (sample size was less than 50, e.g., Field, 2013). Additionally, we also inspected the data for the normality using the histograms and Q–Q plots as well as the skewness and kurtosis values (e.g., Field, 2013). Given that the factorial ANOVA is a robust statistical test that is not sensitive to moderate deviations from the normality, we considered the skewness and kurtosis values between 2 and − 2 within the acceptable range (e.g., Gravetter & Wallnau, 2014; Pek et al., 2018; Trochim & Donnelly, 2006). The sphericity assumption and any relevant corrections did not apply to the current ANOVAs since there were only two levels of the factors. Accordingly, we reported the sphericity assumed results. As the effect size measure, we reported the omega squared (*ω*^2^) based on the previous research suggesting that *ω*^2^ provides an unbiased estimate of the population variances (e.g., Albers & Lakens, 2018; Olejnik & Algina, 2003). We calculated *ω*^2^ with the MOTE calculator using the formula for the factorial repeated-measures ANOVA (Buchanan et al., 2018). We interpreted the *ω*^2^ values of 0.01, 0.06, 0.14 as the small, medium and large effects, respectively (e.g., Field, 2013). Following the main ANOVAs, we planned simple effect analyses with the Bonferroni correction to examine any possible interaction effects. We used an alpha level of 0.05 for all statistical analyses.

We conducted the statistical analyses using IBM SPSS Statistics (Version 27).

#### EEG data analysis

We analyzed the EEG data separately for the verbal and visuospatial task blocks. Following the behavioral analyses, first, we excluded the placement error trials and the trials deviating more than three standard deviations from the individual mean execution time. Then, we conducted EEG data pre-processing.

We filtered the continuous EEG data from each WM task implementing the band-pass filtering from 0.1 to 30 Hz and re-referenced the data to the average mastoid electrodes. Since we were interested in examining the movement re-planning–WM interactions in each WM process, we extracted the stimulus-locked epochs separately for the maintenance and retrieval processes (see Fig. 2). We time-locked the maintenance epochs to the keep/change cue onset with a 100 ms pre-stimulus baseline and a duration of 2500 ms. In the current study, participants started reporting the memory items immediately after the target hit; hence the target hit served as a cue for the WM retrieval. Therefore, we time-locked the retrieval epochs to the target hit with a 100 ms pre-stimulus baseline and a duration of 2000 ms. We considered that the retrieval epochs reflected the cognitive processes related to WM, thus being functionally stimulus-locked as the maintenance epoch. We implemented the Gratton and colleagues’ approach (1983) for the ocular correction and the peak-to-peak moving window approach for the artifact detection on the epoched data. We rejected the epochs containing peak-to-peak amplitudes above the threshold of ± 50 µV within a 200 ms window. Then, we visually double-checked the epochs for the artifacts. If necessary, we interpolated the single bad channels causing the epoch rejection.

After the artifact rejection and channel interpolation, we averaged the maintenance and retrieval epochs across trials separately for the prepared and re-planned movement conditions in the verbal and visuospatial tasks. We obtained individual ERPs for each participant (each experimental condition during the maintenance and retrieval processes). Then, we averaged the individual ERPs across participants to obtain grand-averaged ERPs by excluding the participants losing more than 30% of the epochs for any experimental condition. Accordingly, we included thirty participants for the maintenance process and twenty-eight participants for the retrieval process in the grand averaging. Table 2 shows the average number of epochs entered in the grand-averaged ERPs for each experimental condition during the maintenance and retrieval processes (see Appendix A for the number of epochs entered in the individual ERPs). The average number of epochs even for the re-planned movement condition was compatible with the previous research suggesting that twenty epochs are enough for obtaining reliable P300 waveforms (e.g., Boudewyn et al., 2018; Cohen & Polich, 1997).

**Table 2:** Average number of the epochs entered in the grand-averaged ERPs

Maintenance process				Retrieval process			
Verbal task		Visuospatial task		Verbal task		Visuospatial task	
Prepared	Re-planned	Prepared	Re-planned	Prepared	Re-planned	Prepared	Re-planned
63	26.6	62.9	26.5	61.9	25.7	61.8	26.1

Participants losing more than 30% of the epochs for any experimental condition were excluded from the grand averaging. Accordingly, maintenance process epochs are based on the data from thirty participants, and retrieval epochs are based on the data from 38 participants

To the best our knowledge, the current study was the first to investigate the neurophysiological correlates of the movement re-planning–WM interactions during grasping movements in a dual-task setting. Accordingly, although we expected that the re-planned movements would generate a P300, we analyzed a larger group of the electrodes than the electrodes required for P300 analysis. With this, we assured a comprehensive analysis of the ERPs for the neurophysiological re-planning costs.

First, we determined four region-of-interests (ROI) based on the previous ERP study, investigating the grasping movement–WM interactions (Gunduz Can et al., 2017). The ROIs were systematically aligned across the scalp: left anterior (LA), right anterior (RA), left posterior (LP), right posterior (RP). Each ROI had six electrodes. Electrodes for the LA were Fp1, AF7, AF3, F5, F3, F1. Electrodes for RA were Fp2, AF8, AF4, F6, F4, F2. Electrodes for LP were P5, P3, P1, PO7, PO5, PO3. Electrodes for RP were P6, P4, P2, PO8, PO6, PO4. Based on the previous ERP study and visual inspection of the ERP waveforms, we selected the time window of 200–700 ms.

We conducted the statistical analyses separately for the maintenance and retrieval processes. Mean ERP amplitudes of each ROI within the time window of 200–700 ms entered in the statistical analyses as the dependent variable. We inspected the data for the outliers following the same procedure as the behavioral analyses. Accordingly, we excluded four participants from the maintenance process analysis and two participants from the retrieval process analysis. For each process, we conducted a 2 × 2 × 2 × 2 repeated-measures ANOVA with the factors *WM Task* (verbal vs visuospatial), *Movement Planning* (prepared vs re-planned), *Hemisphere* (left vs right) and *Anterior–posterior Orientation of ROI* (AP; anterior vs posterior).

Second, we determined five midline electrodes for the P300 analysis: Fz, FCz, Cz, CPz, Pz (e.g., Trewartha et al., 2013). We selected the time window of 200–700 ms, considering that P300 is a complex ERP component affected by various factors. We grounded our analyses based on the independent research on action flexibility, grasping movements and WM. However, due to the lack of previous research systematically investigating the neurophysiological correlates of the movement re-planning–WM interactions, we could not ground our analyses directly on a similar approach. For the P300, previous research has suggested different time windows ranging from 200–400 ms (e.g., Nieuwenhuis et al., 2005) to 300–800 ms (e.g., Luck, 1998). Additionally, it has been shown that P300 latency is affected by various factors such as task difficulty (e.g., Kok, 2001; Polich, 2007). In the current study, the cognitive-motor dual-task paradigm included two cognitively demanding tasks, WM tasks and re-planning of the grasp-and-place movement. Accordingly, we conceived that the current dual-task paradigm might delay the P300 latency, thus switching the time window. Moreover, previous ERP research on WM has suggested that verbal and visuospatial information processing has different timings, with a shorter processing time for the visuospatial information than the verbal information (e.g., Bosch et al., 2001; Ruchkin et al., 1992). Although we expected that movement re-planning would generate P300 independent of the WM task, we could not discard the potential effect of the information domain on the ERP effect timing. Therefore, we combined these considerations with the previous research and visual inspection of the ERP waveforms, and selected the time window of 200–700 ms for P300 analyses (e.g., Chase et al., 2011; Krämer et al., 2011).

We conducted the statistical analyses separately for the maintenance and retrieval processes. Mean ERP amplitudes of each electrode within the time window of 200–700 ms entered in the statistical analyses. Following the same procedure for the outlier inspection, we excluded four and three participants, respectively from the maintenance and retrieval process analyses. For each process, we conducted a 2 × 2 × 5 repeated-measures ANOVA with the factors *WM Task* (verbal vs visuospatial), *Movement Planning* (prepared vs re-planned) and *Electrode* (Fz vs FCz vs Cz vs CPz vs Pz).

For all ANOVAs, we checked the normality and sphericity assumptions following the same procedure as the behavioral analyses. Only for the 2 × 2 × 5 repeated-measures ANOVAs, we concerned about the sphericity assumption. We reported the results based on the epsilon (*ε*) value if the sphericity assumption was violated. If the ε value was less than 0.75, we reported the Greenhouse Geisser corrected results. Otherwise, we reported the Huynh-Feldt-corrected results (e.g., Field, 2013). For all other ANOVAs, we reported the sphericity assumed results. We followed the same procedure for the effect size measure, interaction effects, and alpha level as the behavioral analyses.

We performed EEG data pre-processing using custom MATLAB scripts (Version 2016a) combined with the EEGlab toolbox (Version 13.5.4b) and ERPlab toolbox (Version 7.0.0), and conducted the statistical analyses using IBM SPSS Statistics (Version 27).

## Results

### Behavioral results

**Fig. 3:**
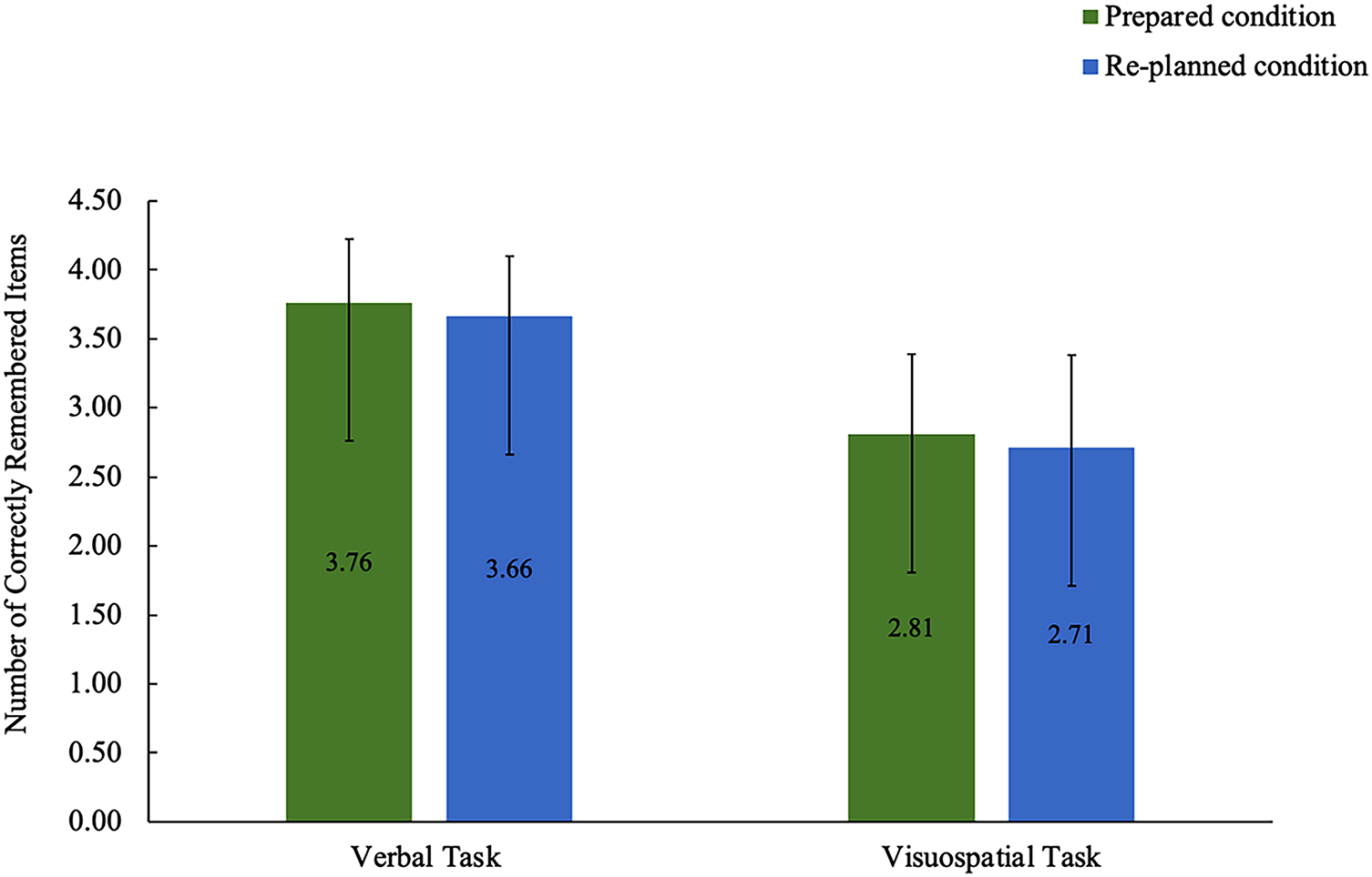
Mean memory performance, in terms of the correctly remembered items, for the verbal and visuospatial tasks in the prepared and re-planned movement conditions. Memory performance was lower for both WM tasks in the re-planned condition than the prepared condition. Error bars represent the standard deviation (calculated on transformed data; “Behavioral data analysis”)

Memory performance for verbal task was on average 3.76 correct letters (SD = 0.47) in prepared movement condition and 3.66 correct letters (SD = 0.43) in re-planned condition. Memory performance for visuospatial task was on average 2.82 correct letters (SD = 0.58) in prepared movement condition and 2.71 correct letters (SD = 0.67) in re-planned condition (see Fig. 3).

The Shapiro–Wilk test revealed that there was no violation to the normality assumption for any experimental condition: Prepared condition (*W* (34) = 0.99, *p* = 0.906) and re-planned condition (*W* (34) = 0.98, *p* = 0.691) in verbal task; prepared condition (*W* (34) = 0.98, *p* = 0.892) and re-planned condition (*W* (34) = 0.95, *p* = 0.122) in visuospatial task. Therefore, we conducted the two-way repeated-measures ANOVA (*WM Task *×* Movement Planning*) and reported the sphericity assumed results. The ANOVA revealed both a main effect of *WM Task*, *F* (1, 33) = 92.41, *p* < 0.001, *ω*^2^ = 0.73, and a main effect of *Movement Planning*, *F* (1, 33) = 12.16, *p* = 0.001, *ω*^2^ = 0.25, indicating large effect sizes for both main effects. Memory performance was significantly lower for the visuospatial task (*M* = 2.76, SD = 0.61) than the verbal task (*M* = 3.71, SD = 0.44) in both the prepared and re-planned conditions (95% CI [0.75, 1.16]). More importantly, memory performance was lower in the re-planned condition (*M* = 3.19, SD = 0.48) than the prepared condition (*M* = 3.28, SD = 0.43) for both the verbal and visuospatial tasks (95% CI [0.04, 0.15]).

The two-way ANOVA did not reveal a significant interaction between *WM Task* and *Movement Planning*, *F* (1, 33) = 0.18, *p* = 0.867, *ω*^2^ = − 0.02.^2^

Movement execution time was on average 2377.76 ms (SD = 491.00) for prepared movements and 2440.69 ms (SD = 546.00) for re-planned movements in verbal task; 2412.47 ms (SD = 590.49) for prepared movements and 2479.68 ms (SD = 585.28) for re-planned movements in visuospatial task.

The Shapiro–Wilk test revealed that there was no violation to the normality assumption for three experimental conditions: Prepared condition (*W* (36) = 0.95, *p* = 0.076) and re-planned condition (*W* (36) = 0.95, *p* = 0.100) in verbal task; prepared condition (*W* (36) = 0.94, *p* = 0.056) in visuospatial task. However, there was violation to the normality assumption for prepared condition (*W* (36) = 0.93, *p* = 0.022) in visuospatial task. Therefore, we further checked the skewness and kurtosis values: Skewness = 0.82 (SE = 0.93) and kurtosis = 0.40 (SE = 0.77). Considering that these values were within the acceptable range, we calculated the two-way repeated-measures ANOVA (*WM Task* × *Movement Planning*) and reported the sphericity assumed results. The ANOVA revealed a main effect of *Movement Planning, F* (1, 35) = 5.28, *p* = 0.028, *ω*^2^ = 0.11, indicating a medium effect size. Namely, movement execution time was longer for the re-planned movements (*M* = 2460.18, SD = 524.81) than the prepared movements (*M* = 2395.11, SD = 511.91) independent of the WM task (95% CI [7.56, 122.57]).

The two-way ANOVA revealed neither a main effect of *WM task*, *F* (1, 35) = 0.38, *p* = 0.566, *ω*^2^ = − 0.02, nor an interaction between the factors, *F* (1, 35) = 0.02, *p* = 0.900, *ω*^2^ = − 0.03.

### ERP results

#### Maintenance process results

For the four-way repeated-measures ANOVA (*WM Task* × *Movement Planning* × *Hemisphere* × *AP*), Table 3 shows the ERP amplitudes and standard deviations for each experimental condition in each hemisphere and anterior and posterior orientation.

**Table 3:** Four-way repeated-measures ANOVA for the maintenance process ERP amplitudes and standard deviations for each experimental condition

	* M*	SD
Prepared verbal task–left hemisphere anterior	1.55	3.46
Prepared verbal task–left hemisphere posterior	− 3.73	4.69
Prepared verbal task–right hemisphere anterior	1.31	4.40
Prepared verbal task–right hemisphere posterior	− 5.08	4.72
Re-planned verbal task–left hemisphere anterior	2.61	4.08
Re-planned verbal task–left hemisphere posterior	− 2.68	5.30
Re-planned verbal task–right hemisphere anterior	2.70	4.62
Re-planned verbal task–right hemisphere posterior	− 4.02	4.79
Prepared visuospatial task–left hemisphere anterior	2.51	3.21
Prepared visuospatial task–left hemisphere posterior	− 4.14	5.50
Prepared visuospatial task–right hemisphere anterior	1.92	3.96
Prepared visuospatial task–right hemisphere posterior	− 5.85	5.85
Re-planned visuospatial task–left hemisphere anterior	2.97	3.39
Re-planned visuospatial task–left hemisphere posterior	− 2.98	6.34
Re-planned visuospatial task–right hemisphere anterior	2.08	4.83
Re-planned visuospatial task–right hemisphere posterior	− 4.63	6.66

ERP amplitudes are based on the four ROIs between 200 and 700 ms

The Shapiro–Wilk test revealed that there was no violation to the normality assumption for any experimental conditions (all *W* (26) > 0.93, all *p* > 0.076). Therefore, we conducted the four-way repeated-measures ANOVA and reported the sphericity assumed results. The ANOVA revealed a main effect of *Movement Planning* between 200 and 700 ms, *F* (1, 25) = 5.78, *p* = 0.024, *ω*^2^ = 0.16, indicating a large effect size. ERP amplitudes were more positive in the re-planned condition (*M* = − 0.50, SD = 3.70) than the prepared condition (*M* = − 1.44, SD = 3.24) at all ROIs independent of the WM task (95% CI [0.14, 1.75]).

**Fig. 4:**
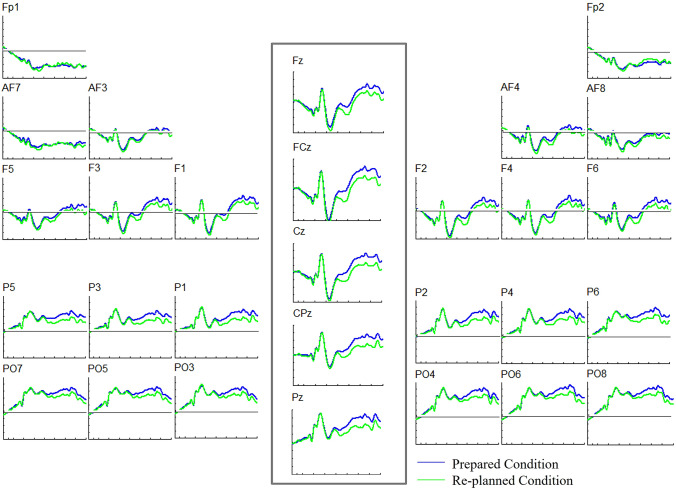
Grand average ERP waveforms during the maintenance process. ERPs are superimposed for the prepared movement trials (blue line) and the re-planned movement trials (green line) in the verbal and visuospatial tasks. Six electrodes from each ROI are shown and arrayed from left to right and from anterior to posterior as they were positioned on the scalp. Five midline electrodes are shown and arrayed from anterior to posterior. Negativity is plotted upwards. Stimulus onset occurred at 0 ms as the keep/change cue onset

The ANOVA did not reveal any other significant results concerning the factors of interest, *WM Task* and *Movement Planning* (all *F* (1, 25) < 1.96, all *p* > 0.176, all *ω*^2^ < 0.03). Accordingly, these results indicated that prepared and re-planned movements generated differentiating ERPs independent of the WM task, pointing towards a re-planning effect between 200 and 700 ms (see Fig. 4 for the ERP plots).

For the three-way repeated-measures ANOVA (*WM Task* × *Movement Planning* × *Electrode*), Table 4 shows the ERP amplitudes and standard deviations for each experimental condition in each electrode.

**Table 4:** Three-way repeated-measures ANOVA for the maintenance process ERP amplitudes and standard deviations for each experimental condition

	* M*	* SD*
Prepared verbal task–electrode Fz	− 0.07	4.45
Prepared verbal task–electrode FCz	− 0.70	4.51
Prepared verbal task–electrode Cz	− 0.70	4.37
Prepared verbal task–electrode PCz	− 1.82	5.04
Prepared verbal task–electrode Pz	− 3.45	5.25
Re-planned verbal task–electrode Fz	1.65	4.34
Re-planned verbal task–electrode FCz	1.22	4.17
Re-planned verbal task–electrode Cz	0.82	3.78
Re-planned verbal task–electrode PCz	− 0.45	4.39
Re-planned verbal task–electrode Pz	− 2.41	5.11
Prepared visuospatial task–electrode Fz	1.27	4.90
Prepared visuospatial task–electrode FCz	0.66	5.15
Prepared visuospatial task–electrode Cz	0.71	4.90
Prepared visuospatial task–electrode PCz	− 1.20	5.71
Prepared visuospatial task–electrode Pz	− 3.50	6.59
Re-planned visuospatial task–electrode Fz	2.18	5.53
Re-planned visuospatial task–electrode FCz	2.00	5.70
Re-planned visuospatial task–electrode Cz	2.21	5.79
Re-planned visuospatial task–electrode PCz	0.20	6.67
Re-planned visuospatial task–electrode Pz	− 2.07	7.36

ERP amplitudes are based on the five electrodes between 200 and 700 ms

The Shapiro–Wilk test revealed that there was no violation to the normality assumption for any experimental conditions (all *W* (26) > 0.93, all *p* > 0.061). Therefore, we conducted the three-way repeated-measures ANOVA. Only for the factor *Electrode,* sphericity assumption was concerned. Mauchly’s test revealed the violation to the sphericity assumption for the main effect of *Electrode* (*χ*^2^ (9) = 0.04, *p* < 0.001) as well as the interactions between *WM Task* and *Electrode* (*χ*^2^ (9) = 0.01, *p* < 0.001), *Movement Planning* and *Electrode* (*χ*^2^ (9) = 0.02*, p* < 0.001) and *WM Task*, *Movement Planning* and *Electrode* (*χ*^2^ (9) = 0.02*, p* < 0.001). Therefore, we reported the Greenhouse–Geisser corrected results concerning the factor *Electrode* (*ε* = 0.39 for the main effect; *ε* = 0.31 for *WM Task* × *Electrode*; *ε* = 0.36 for *Movement Planning* × *Electrode*; *ε* = 0.36 for *WM Task* × *Movement Planning* × *Electrode*), while we reported the sphericity assumed results concerning the factors *WM Task* and *Movement Planning.*

Similar to the four-way repeated-measures ANOVA, the three-way ANOVA also revealed a main effect of *Movement Planning* between 200 and 700 ms, *F* (1, 25) = 8.96, *p* = 0.006, *ω*^2^ = 0.23, indicating a large effect size. ERP amplitudes were more positive in the re-planned condition (*M* = 0.53, SD = 4.33) than the prepared condition (*M* = − 0.88, SD = 4.47) at all electrodes independent of the WM task (95% CI [0.44, 2.38]).

**Fig. 5:**
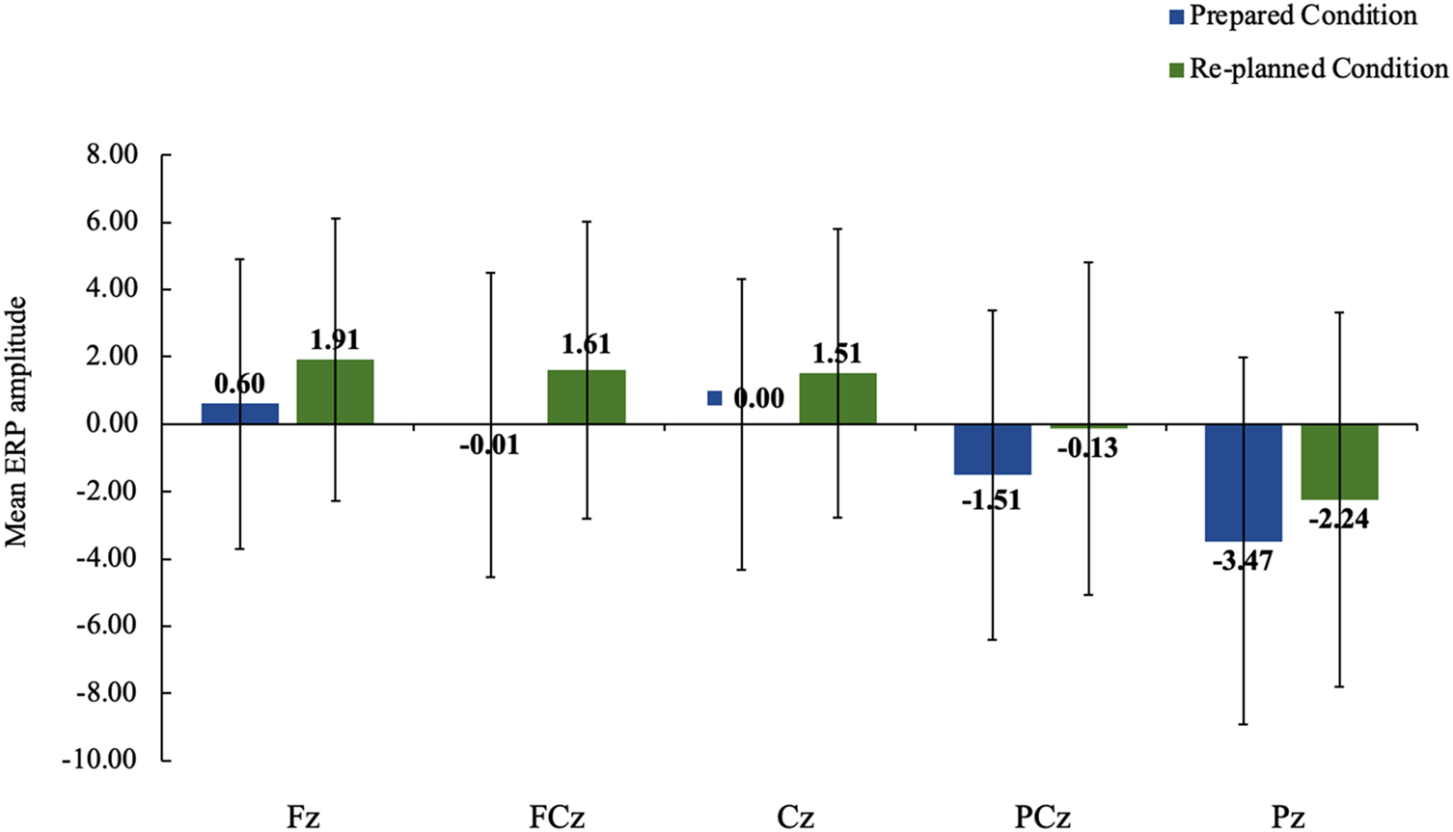
Bar chart represents the ERP amplitudes for the prepared movement trials (blue bars) and the re-planned movement trials (green bars) at each midline electrode during the maintenance process. In line with the 2 × 2 × 5 repeated-measures ANOVA (*WM Task* × *Movement Planning* × *Electrode*), bar chart also shows the more positive amplitudes for the re-planned movements than the prepared movements between 200 and 700 ms at each electrode

**Fig. 6:**
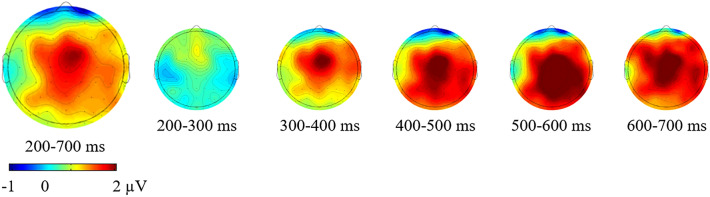
Topographic maps represent the re-planned movement-minus-prepared movement difference potentials during the maintenance process. The first map shows the re-planning effect between 200 and 700 ms, and five 100 ms maps show the development of the re-planning effect

We interpreted the positive slow wave for the re-planned movements as a P300 component considering the scalp topography and timing, which were maximally localized over the midline electrodes and increased in positivity from the anterior to posterior scalp regions between 200 and 700 ms (see Fig. 4 for the ERP plots for midline electrodes). Based on the ERP plots for midline electrodes, we obtained a bar chart that represented the ERP amplitude differences between the prepared and re-planned movements at each electrode, thus providing better visualization of the P300 (see Fig. 5 for the bar chart). Moreover, we obtained five 100-ms topographic maps, which represented the re-planned movement-minus-prepared movement difference potentials, to trace the development of the re-planning effect over time (see Fig. 6 for the topographic maps). These topographic maps also presented that the re-planning effect emerged as an anterior-focused positivity and continued as a posterior-focused positivity over time, consistent with the P300 result.^3^

The three-way ANOVA did not reveal any other significant results concerning the factors of interest, *WM Task* and *Movement Planning* (all *F* (1, 25) < 1.65, all *p* > 0.167, all *ω*^2^ < 0.02).

#### Retrieval process results

For the four-way repeated-measures ANOVA (*WM Task* × *Movement Planning* × *Hemisphere* × *AP*), the Shapiro–Wilk test revealed that there was no violation to the normality assumption for any experimental conditions (all *W* (26) > 0.92, all *p* > 0.051), except the following: In verbal task, prepared condition at left anterior ROI (*W* (26) = 0.91, *p* = 0.029), and re-planned condition at left anterior ROI (*W* (26) = 0.85, *p* = 0.002) and right anterior ROI (*W* (26) = 0.90, *p* = 0.016); in visuospatial task, prepared condition at left posterior ROI (*W*(26) = 0.90, *p* = 0.014). Skewness and kurtosis values were within the acceptable range, with the largest skewness = − 1.24 (SE = 0.46) and kurtosis = 1.68 (SE = 0.89) for prepared condition in visuospatial task. Therefore, we conducted the four-way repeated-measures ANOVA and reported the sphericity assumed results. ANOVA did not reveal any significant results between 200 and 700 ms concerning the factors of interest, *WM Task* and *Movement Planning* (see Table 5 for the ANOVA results).

**Table 5:** Four-way repeated-measures ANOVA for the retrieval process

	*df*	*F*	*p*	* ω*^2^
WM	25	2.74	0.111	0.06
Planning	25	1.66	0.209	0.02
WM × Planning	25	0.65	0.428	0.02
WM × H	25	0.21	0.649	− 0.03
Planning × H	25	0.49	0.489	− 0.02
WM × Planning × H	25	0.01	0.946	− 0.04
WM × AP	25	0.37	0.847	− 0.04
Planning × AP	25	0.25	0.621	− 0.03
WM × Planning × AP	25	1.58	0.221	0.02
WM × H × AP	25	0.50	0.486	− 0.02
Planning × H × AP	25	0.00	0.952	− 0.04
WM × Planning × H × AP	25	0.05	0.829	− 0.04

The results only for the factors of interest

*WM* WM task, planning movement planning, *H* hemisphere, *AP* anterior–posterior orientation

**Fig. 7:**
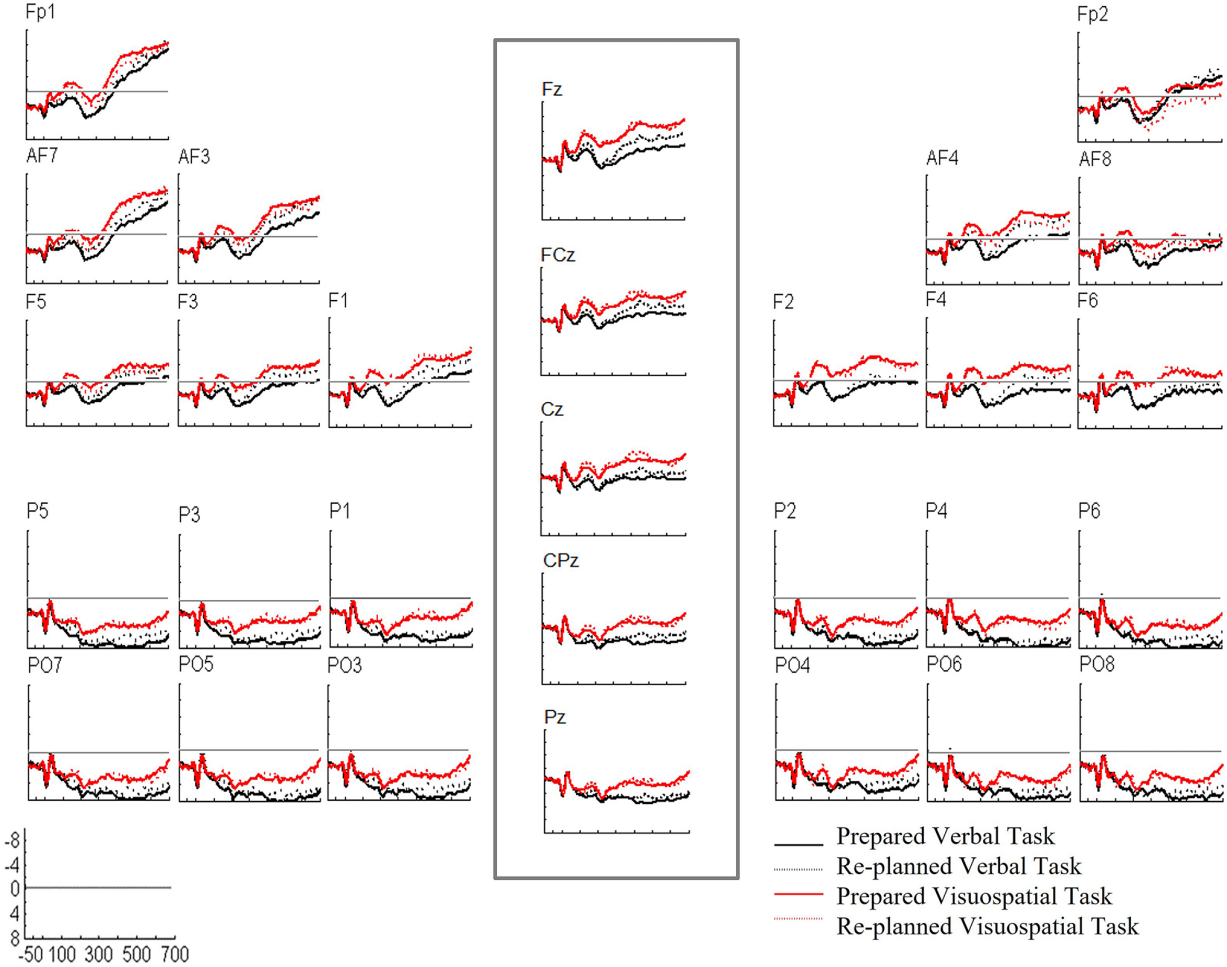
Grand average ERP waveforms during the retrieval process. ERPs are superimposed for the prepared movement trials (black solid line) and the re-planned movement trials (black dotted line) in the verbal task, and the prepared movement trials (red solid line) and the re-planned movement trials (red dotted line) in the visuospatial task. Six electrodes from each ROI are shown and are arrayed from left to right and from anterior to posterior as they were positioned on the scalp. Five midline electrodes are shown and arrayed from anterior to posterior. Negativity is plotted upwards. Stimulus onset occurred at 0 ms as the target hit

For the three-way repeated-measures ANOVA (*WM Task* × *Movement Planning* × *Electrode*), the Shapiro-Wilk test revealed that there was no violation to the normality assumption for any experimental conditions (all *W* (25) > 0.93, all *p* > 0.071). Therefore, we conducted the three-way repeated-measures ANOVA. Mauchly’s test revealed the violation to the sphericity assumption for the main effect of *Electrode* (*χ*^2^ (9) = 0.02, *p* < 0.001) as well as the interactions between *WM Task* and *Electrode* (*χ*^2^ (9) = 0.06, *p* < 0.001), *Movement Planning* and *Electrode* (*χ*^2^ (9) = 0.02, *p* < 0.001) and *WM Task*, *Movement Planning* and *Electrode* (*χ*^2^ (9) = 0.02, *p* < 0.001). Therefore, we reported the Greenhouse–Geisser corrected results concerning the factor *Electrode* (*ε* = 0.39 for the main effect; *ε* = 0.43 for *WM Task* × *Electrode*; *ε* = 0.40 for *Movement Planning* × *Electrod*e; *ε* = 0.39 for *WM Task* × *Movement Planning* × *Electrode*), while we reported sphericity assumed results concerning the factors *WM Task* and *Movement Planning.* Similar to the four-way repeated-measures ANOVA, three-way ANOVA also did not reveal any significant results between 200 and 700 ms concerning the factors of interests, *WM Task* and *Movement Planning* (see Table 6 for the ANOVA results and Fig. 7 for the ERP plots).

**Table 6:** Three-way repeated-measures ANOVA for the retrieval process

	*df*	*F*	*p*	* ω*^2^
WM	24	2.65	0.117	0.06
Planning	24	2.41	0.134	0.05
WM × Planning	24	0.05	0.830	− 0.04
WM × E	24	0.36	0.670	− 0.03
Planning × E	24	0.19	0.781	− 0.04
WM × Planning × E	24	0.78	0.456	− 0.01

The results only for the factors of interest. Results concerning the factor *Electrode* are based on the Greenhouse Geisser correction. Other results are sphericity assumed

*WM* WM task, planning movement planning, *E* electrode

## Discussion

Here, we examined the neurophysiological correlates of the movement re-planning–WM interactions by focusing on the grasping movements and separate WM domains (verbal, visuospatial) and processes (maintenance, retrieval). We combined a cognitive-motor dual-task paradigm with an EEG setting. Participants completed a WM task (verbal and visuospatial versions) concurrently with a manual task. The manual task required performing a grasp-and-place movement by keeping the initial movement plan (prepared movement condition) or changing it for reversing the movement direction (re-planned movement condition). In line with our hypotheses, behavioral analyses showed a lower memory performance for the verbal and visuospatial tasks in the re-planned condition than the prepared condition. ERP analyses showed a larger positive slow wave for the re-planned movements than the prepared movements only during the maintenance process in both WM tasks. There was no ERP difference between the prepared and re-planned movements during the retrieval process. We interpret these findings indicating that re-planning the grasp-and-place movement interfered at least with the maintenance of the verbal and visuospatial domains, resulting in the domain-general, process-specific re-planning costs.

### Behavioral re-planning costs

The primary behavioral finding is that memory performance was lower in the re-planned condition than the prepared condition independent of the WM task. Re-planning the grasp-and-place movement interfered with memorizing the verbal and visuospatial information to a similar degree, hence decreasing the memory performance for the verbal and visuospatial tasks. We interpret the memory performance decrease for both tasks as the behavioral domain-general re-planning costs. This finding replicates the behavioral findings by Spiegel and colleagues (2013), who also showed the movement re-planning costs for both the verbal and visuospatial domains.

In the current study, during both the prepared and re-planned movements, satisfying the desired action outcome, i.e., placing the sphere on the correct motor target, required accessing the pointing direction of the arrow, interpreting the meaning of the auditory tone and deciding whether to change the initial movement plan. In addition, during the re-planned movements, upon receiving the unexpected change cue, it also required understanding the mismatch between the initial plan and the desired action outcome, i.e., reversed movement direction. As a result, during the re-planned movements, satisfying the desired action outcome also required canceling the initial plan, inhibiting the movement planned towards the pointed motor target, comprehending the reversed pointing direction of the arrow (opposite motor target), and selecting and preparing an appropriate new plan based on the reversed movement direction. Accordingly, re-planned movements demanded additional cognitive operations for adapting to the changing action demand. Previous research has also suggested the additional cognitive operations involved in the movement re-planning and further shown that movement re-planning recruits attention and WM resources, for example, for evaluating the mismatch between the initial plan and desired action outcome (e.g., Trewartha et al., 2013) and reconfiguring the stimulus–response pairs (e.g., Mostofsky & Simmonds, 2008; Yamanaka & Nozaki, 2013).

We argue that movement re-planning shares the capacity-limited cognitive resources with WM due to these additional cognitive operations. Consequently, when completed concurrently with WM tasks, it draws on the shared resources and leaves less cognitive resources for WM, thus leading the interference. Moreover, considering the re-planning interference with both the verbal and visuospatial domains in the current study, we argue that movement re-planning draws on a cognitive resource common for both WM domains. We propose that this common resource is the domain-general attention mechanisms.

Different WM models have discussed the role of the domain-general attention mechanisms in WM. For example, the multi-component model (Baddeley & Hitch, 1974) has proposed that the central executive is the domain-general attention mechanism that is involved in various executive tasks, such as coordinating the concurrent tasks or maintaining the verbal and visuospatial information under cognitively demanding task situations (e.g., Allen et al., 2017; Baddeley, 1996). Similarly, the state models have also proposed that domain-general attention mechanisms maintain any information (e.g., Barrouillet et al., 2007; Cowan, 1999; Oberauer, 2002). For example, the time-based resource sharing model (Barrouillet et al., 2007) has proposed that information is maintained in WM through attentional refreshing, which brings attention to to-be-remembered information. However, any other task also uses the same attention mechanisms. Therefore, any concurrent task captures the same attention mechanisms that also maintain the information in WM, thus interfering with the maintenance process. Moreover, such interference depends on the difficulty of the concurrent task. Cognitively demanding concurrent tasks capture the attention mechanisms longer, hence leading the larger interference.

The current cognitive-motor dual-task paradigm required concurrent completion of movement re-planning and WM tasks. In the current paradigm, the domain-general attention mechanisms changed the initial movement plan (movement re-planning itself), memorizing the verbal and visuospatial information in the presence of movement re-planning (WM tasks itself), and coordinated the concurrent movement re-planning and WM tasks (dual-task). Consequently, we argue that movement re-planning with additional cognitive operations acted as a distracter and drew on the same domain-general but capacity-limited attention mechanisms. With this, movement re-planning left fewer cognitive resources for WM, thus interfering with memorizing the verbal and visuospatial information. As a result, memory performance decreased for the verbal and visuospatial tasks. The idea that movement re-planning increases the demand for attention mechanisms is also consistent with the previous research (e.g., Caljouw et al., 2011; Gritsenko et al., 2009; Verbruggen et al., 2010).

Another finding regarding the memory performance was that participants performed worse for the visuospatial task than the verbal task in the prepared and re-planned conditions. This finding is consistent with a previous ERP study, which compared the memory performances for the verbal and visuospatial tasks between a baseline single-task condition (only WM task) and a dual-task condition (WM task and manual task; Gunduz Can et al., 2017). In the single-task, memory performance was on average 4 items for the verbal task and 3.7 items for the visuospatial task, consistent with the proposed WM capacity (3–4 items on average; Cowan, 2001). In contrast, in the dual-task, memory performance was on average 4 items for the verbal task and 3.1 items for the visuospatial task. Memory performance for the visuospatial but not for the verbal task decreased in the dual-task than the single-task. Unlike the current re-planning costs, the previous study showed that performing a merely added grasp-and-place movement (without movement re-planning) interfered only with the visuospatial but not with the verbal domain, resulting in the domain-specific execution costs (Gunduz Can et al., 2017). Given that the current study always included the dual-task, we interpret the lower memory performance for the visuospatial task, particularly in the prepared condition, as the selective interference of the movement execution with the visuospatial domain.

Besides the cognitive re-planning costs, movement re-planning also entailed motor costs. That is, independent of the WM task, movement execution time was longer for the re-planned movements than the prepared movements. The longer execution times for the re-planned movements are consistent with the previous research suggesting that changing the prepared movement plans takes longer than executing the movement as initially planned (e.g., Barrett & Glencross, 1989; Hughes et al., 2012; Oostwoud Wijdenes et al., 2013). For example, Hughes and colleagues (2012) showed that the condition that required correcting a grasp posture by changing a prepared movement plan resulted in longer execution times than the condition that required keeping the grasp posture. Accordingly, we argue that changing the prepared movement plan entailed additional motor operations, such as changing the muscle groups involved in reversing the movement direction, resulting in longer execution times (e.g., Elliott et al., 2017).

In summary, the current behavioral findings indicate that re-planning the grasp-and-place movement interferes with memorizing the verbal and visuospatial information to a similar degree, thus decreasing the memory performance for the verbal and visuospatial tasks. That is, movement re-planning entails behavioral domain-general re-planning costs. We propose that the shared capacity-limited cognitive resources, such as domain-general attention mechanisms, involved both in movement re-planning and WM are the potential source of the domain-general re-planning costs.

### Neurophysiological re-planning costs

The primary neurophysiological finding is that prepared and re-planned movement conditions generated differentiating ERPs during the maintenance process independent of the WM task. ERPs for the prepared and re-planned movements started to differ about 200 ms following the keep/change cue onset and continued until 700 ms over the anterior and posterior recording sites during the maintenance process in the verbal and visuospatial tasks. These ERP differences showed a larger positive slow wave for the re-planned movements than the prepared movements. We interpret the larger positivity as a P300 component considering the scalp topography and timing (with a centroparietal maximum between 200 and 700 ms). These findings indicate that movement re-planning interferes at least with the maintenance of the verbal and visuospatial domains, resulting in the neurophysiological re-planning costs.

P300 has been considered a specific ERP component associated with the task context updating, mainly updating of WM upon receiving new task-relevant information. In this regard, one of the classical explanations of the P300 has been the ‘context updating theory’ (Donchin & Coles, 1988). According to this theory, the brain constructs a mental representation of the current task context. When new task-relevant information appears, it may change the task context and induce a mismatch between the current mental representation and the new context; hence new information may require updating the mental representation. Therefore, when the new information appears, it is necessary to evaluate whether it induces a mismatch. In the case of a mismatch, the current mental representation is updated according to the new information. P300 reflects the cognitive processes, particularly WM, involved in evaluating the new information and updating the mental representation accordingly. The context updating theory focuses solely on the stimulus information as the key for changing the task context and excludes any information related to the response.

In the reformulated version of the context updating theory, Verleger and colleagues (2005) have proposed that stimulus is not separate from the response. Therefore, any stimulus information includes also the response information to a certain extent. For example, a particular stimulus indicates a specific response and creates a stimulus–response pair, i.e., task context. Consequently, a mental representation of this stimulus–response pair is constructed. When a new stimulus appears, it indicates a new response, thus creating another stimulus–response pair, i.e., changed task context. As a result, the new stimulus induces a mismatch between the current mental representation and the new context. In this case, the current mental representation is updated based on the new stimulus information, mainly its pairing with the response. According to the reformulated version of the context updating theory, P300 reflects the cognitive processes involved in evaluating the new information based on the stimulus–response pair and updating the mental representation accordingly.

Action flexibility research has linked the context updating, mainly through stimulus–response pair, with movement re-planning and suggested that P300 reflects the cognitive processes involved in context updating during movement re-planning (e.g., Chase et al., 2011; Fleming et al., 2009; Krämer et al., 2011; Krigolson & Holyroyd, 2008; Krigolson et al., 2008; Randall & Smith, 2011; Trewartha et al., 2013; Ullsperger et al., 2014a, b). During movement re-planning tasks, particular stimulus–response pairs represent the task context, based on which the internal action representations, including the movement plans and parameters, are constructed. After the response is planned but not executed yet, a new stimulus such as a change cue appears and indicates a new response, thus changing the task context. Consequently, the new stimulus, mainly through its pairing with the new response, also induces mismatch between the current action representations and the new context. Therefore, action representations, including the movement plans and parameters, are updated accordingly. Notably, during movement re-planning, the stimulus information (e.g., change cue) influences the response (e.g., planned response), thus influencing the task context (e.g., stimulus–response pair) and its mental representation (e.g., internal action representations). Therefore, it follows that context updating allow for movement re-planning when new stimulus information changes the current response and ask for an alternative new movement plan.

In line with the link between the context updating and movement re-planning, it has been shown that P300 is associated with evaluating the mismatch between the internal action representations and the task context, thus updating the action representations (e.g., Krigolson et al., 2008; Ullsperger et al., 2014a, b). Specifically, P300 is associated, for example, with inhibiting the planned response and reconfiguring the stimulus–response pairs during movement re-planning (e.g., Krämer et al., 2011; Randall & Smith, 2011). For example, Trewartha and colleagues (2013) examined the P300 during movement re-planning based on the context updating, mainly comparing the elderly with the younger adults. Participants performed a keypress task in which the trials included either valid or invalid stimulus–response pairs. After planning the first response, participants executed an alternative response in the invalid trials. Hence, they had to re-plan the prepared response. The authors showed that invalid trials generated a larger P300 than valid trials in the elderly and younger adults. Moreover, only in the younger adults, efficient re-planning was associated with the faster response times and the larger P300. Accordingly, the authors suggested that efficient re-planning, operationalized with faster response times and larger P300, is associated with better context updating ability, mainly due to the better WM capacity (but see Rac-Lubashevsky et al., 2019). Hence, the elderly with the impaired WM capacity, thus impaired context updating ability, demonstrated inefficient re-planning and generated smaller P300 in the invalid trials.

Similarly, Fleming and colleagues (2009) linked the context updating and P300 with movement re-planning, mainly comparing the instructed and freely chosen actions. In a keypress task, participants planned a left or right response based on an arrow pointing towards the left or right target (instructed) or pointing towards both (freely chosen). As in the current study, the keep/change cue was presented after the participants planned but did not execute the response yet. While the keep cue asked for executing the planned response, the change cue asked for reversing the response direction (i.e., re-planning). The authors showed that the change cue generated a larger P300 than the keep cue, particularly during the instructed responses. That is, re-planning instructed responses was associated with P300 better. The authors discussed this finding in line with the context updating theory and suggested that instructed responses required more commitment, thus being less flexible and adaptable. Consequently, instructed responses demanded more context updating than the freely chosen responses and generated larger P300 when re-planned.

Based on the previous research, we suggest that the current P300 reflects the context updating during movement re-planning, mainly through stimulus–response pair. In the current study, an arrow pointed towards the left or right motor target, thus indicating the movement direction, i.e., desired action outcome. Hence, the arrow served as a stimulus and paired with a specific response (left or right movement). This stimulus–response pair created a task context, associating with the internal action representations stored in WM. In some trials, an unexpected auditory tone appeared as a change cue and reversed the movement direction, i.e., changed action outcome. Hence, the auditory tone served as a new stimulus (by reversing the pointing direction of the arrow), pairing with a new response (right movement instead of left or vice versa). Consequently, the auditory tone changed the stimulus–response pair, thus changing the task context. Therefore, it also induced mismatch between the current action representations and the new context, requiring updating the action representations in WM. During movement re-planning, this update was achieved by changing the initial movement plan favoring an alternative new plan. Accordingly, context updating allowed adapting the planned movements to the changing action demands during re-planned grasp-and-place movements. Consequently, re-planned grasp-and-place movements generated a larger P300 than the prepared movements since there was no context updating during prepared movements.

The link between the context updating, particularly the updating of WM with new stimulus–response pair, and movement re-planning also conforms to the motor control research suggesting the functional role of WM in manual action control. For example, WM stores the task-related target information for the upcoming movement (e.g., Hesse & Franz, 2009, 2010; Hesse et al., 2016; Kohler et al., 1989; for a review, see Schenk & Hesse, 2018). Importantly, WM selects and prepares the movement plans, and changes them favoring the alternative new plans when necessary (e.g., Fournier et al., 2014; Gallivan et al., 2016). Accordingly, we suggest that this link, mainly through WM, also supports the current behavioral re-planning costs. It is intuitive to think that movement re-planning drew on the capacity-limited WM resources for updating the action representations and left fewer resources for the maintenance of the verbal and visuospatial information. Consequently, movement re-planning interfered with the verbal and visuospatial domains during maintenance. If the maintenance of the verbal and visuospatial information was intact, we should have seen the differentiating slow waves for the verbal and visuospatial domains. Based on the widely reported slow waves, we should have seen the left anterior negative slow wave for the verbal domain maintenance (e.g., Ruchkin, Berndt, Johnson, et al., 1997a, b; Ruchkin et al., 1990, 1992) and the (right) posterior negative slow wave for the visuospatial domain maintenance (e.g., Geffen et al., 1997; Löw et al., 1999; Ruchkin et al., 1992; Ruchkin, Johnson, Grafman, et al., 1997a, b).

Consequently, we propose that re-planned grasp-and-place movements disrupted the maintenance of the verbal and visuospatial domains. The decreased memory performance for the verbal and visuospatial tasks as well as the longer movements times and the larger P300 for the re-planned movements (without expected ERPs for WM maintenance) demonstrated this disruption. However, our data did not show a significant correlation between the behavioral memory performance and P300 based on the differences between the prepared and re-planned movement conditions. Therefore, future research should investigate the potential (causal) relationship between behavioral memory performance and P300.

We cannot rule out the possibility in the current study that the infrequent change cue affected the P300. One of the typical experimental tasks for the P300 investigation is the oddball paradigms, in which the infrequent target stimuli are embedded within the frequent nontarget stimuli. In the oddball paradigms, the infrequent target stimuli generate P300, which show the inverse relationship between the stimulus probability and the P300 amplitude. The less probable the stimulus is, the larger the P300 (e.g., Donchin & Coles, 1988; Polich, 2007). Similarly, in the current dual-task paradigm, the change cue was inherently less frequent, thus less probable, than the keep cue. However, we would like to emphasize that the current paradigm is compatible with the action flexibility research investigating the movement re-planning and its association with P300 (e.g., Chase et al., 2011; Hughes et al., 2012; Hughes & Seegelke, 2013; Krämer et al., 2011; Krigolson & Holyroyd, 2008; Randall & Smith, 2011; Trewartha et al., 2013). The infrequent change cue in such investigations prevents participants from guessing the upcoming movement condition, thus making them plan the movement as if no re-planning would be required. With this, it is possible to investigate how and to what extent participants adapt the planned movements to the changing action demands through movement re-planning. Therefore, such investigations generally include the 3:1 or 4:1 ratio among the keep/change cues, i.e., prepared/re-planned movements (for a review, see Smeets et al., 2016). Similarly, we included 70 trials with the keep cue (prepared movement condition) and 30 trials with the change cue (re-planned movement condition). As a result, we aimed for avoiding the high number of trials, thus preventing the fatigue in the dual-task, but still having enough trials in the re-planned condition, mainly for ERP analyses.

Admitting the potential effect of the infrequent change cue on the current P300, we argue that the current P300 reflects the context updating during movement re-planning. First, as aforementioned, the current dual-task paradigm aligns with the previous research investigating the movement re-planning with the frequent keep cue/infrequent change cue ratios. Notably, the current paradigm also demonstrates the findings consistent with the previous research linking the context updating with movement re-planning and suggesting the P300 reflecting this link. Second, ERP findings, i.e., larger P300 for the re-planned movements, also conforms to the behavioral findings showing the decreased memory performance for the verbal and visuospatial tasks in the re-planned condition. We interpret these findings indicating that movement re-planning interferes with the maintenance of the verbal and visuospatial information due to the shared cognitive resources. Third, the distribution of the current P300 is also in line with the previous neuroimaging research suggesting the fronto-temporal-parietal network, such as the inferior frontal cortex (e.g., Mars et al., 2007; Neubert et al., 2010), the pre-supplementary motor area (e.g., Mars et al., 2009; Neubert et al., 2011) and the dorsal premotor cortex involved (e.g., Hartwigsen & Siebner, 2015; Hartwigsen et al., 2012) in the movement re-planning.

Furthermore, we have indirectly tested the impact of tone frequency. In a previous ERP study, we implemented the same paradigm as here, including the auditory tone (Gunduz Can et al., 2017). We kept the auditory tone in the previous study to ensure comparability with the previous behavioral studies (Spiegel et al., 2013, 2014). Although there was the auditory keep/change cue, participants did not re-plan the grasp-and-place movement in Gunduz Can et al. (2017). Instead, participants performed the movement always as planned. We analyzed the previous data to examine whether the mere presence of infrequent auditory tones would generate a P300 given that there was no need for movement re-planning. We followed the same analysis steps as the current study and conducted a 2 × 2 × 5 repeated-measures ANOVA with the factors *WM Task* (verbal vs visuospatial), *Movement Planning* (prepared vs re-planned) and *Electrode* (Fz vs FCz vs Cz vs CPz vs Pz) with a time window of 200–700 ms. This ANOVA did not reveal any significant result, indicating that the mere presence of infrequent auditory tones is unlikely to generate a P300 during the maintenance of the verbal and visuospatial domains.

The absence of the re-planning effect during the retrieval process seems to indicate that movement re-planning interferes only with the maintenance process. Here, we highlight some points before making the conclusion about the retrieval process and process specificity of the re-planning costs. In the current study, we adapted a well-established cognitive-motor dual-task paradigm from the previous behavioral studies showing the behavioral re-planning costs for WM (Spiegel et al., 2012, 2013, 2014). We demonstrated this paradigm’s EEG adaptability also in our previous ERP study (Gunduz Can et al., 2017). Considering the importance of the replication in research, we kept the current dual-task paradigm as constant as possible with the previous studies. However, we acknowledge that this paradigm might have created some limitations for investigating the retrieval process.

In the current paradigm, participants memorized the memory items while placing the sphere on the motor target. The sphere placement ended the grasp-and-place movement, i.e., target hit, and served as a cue for WM retrieval. Therefore, we assumed that all participants started reporting the memory items in a comparably similar fashion across the trials. Consequently, we time-locked the retrieval epochs to the target hit, considering that this reflects the cognitive processes and underlying neurophysiological activity related to the retrieval. We admit that this time-locking might have led the jittering across the trials among participants and reduced the ERP effect. Therefore, the target hit might not have been the perfect time for investigating the retrieval. However, we still argue that it was the best estimate available while keeping the current paradigm constant with regard to the previous studies. Moreover, we argue that the current lack of re-planning effect is not the sole result of the jittering, mainly given that the current paradigm required participants to start reporting the memory items, thus engaging in the retrieval, immediately after the target hit across the trials.

Additionally, we admit the limitation of the response form and modality for the retrieval. Participants reported memory items written in a free recall format in the current study. We analyzed the final memory performance for the behavioral data, i.e., the items remembered. However, we analyzed only a few seconds for the ERPs. The primary reason for this short time interval was to prevent the movement artifacts that would have been larger during longer time intervals. However, this short interval might have limited the analysis of the whole retrieval effort. Here, we argue that it was essential to see the memory performance decrease as the number of correctly remembered items and relate it to the neurophysiological activity. Accordingly, the current free recall paradigm offered a suitable option, consistent also with the previous studies. Alternatively, future research should consider implementing recognition paradigms such as delayed match-to-sample task, consistent with the previous ERP research on WM (e.g., Löw et al., 1999; Ruchkin et al., 1990, 1992).

The current manual response modality might have possibly concealed the potential re-planning effect due to the motor-related cortical activity common for the hand movements required for grasp-and-place movement and written WM report (e.g., Westerholz et al., 2013, 2014). Future research should focus on different WM response modalities, such as a spoken report, to investigate the reason for the absence of the re-planning effect, i.e., lack of re-planning interference or WM response modality.

We aimed for determining the source of the movement re-planning–WM interactions and the underlying neurophysiological activity. The current findings point to the maintenance process as the source of the interaction. One possibility is that movement re-planning actually interacts only with the maintenance. An alternative possibility is that the movement re-planning interacts with the maintenance since the change cue indicating the context updating appeared there. Therefore, before concluding about the movement re-planning–WM interactions and the process specificity of the re-planning costs, it is essential to investigate whether the comparable findings are obtained when the change cue appeared during the retrieval. Again, we highlight the current study's importance as the initial investigation of the neurophysiological correlates of the movement re-planning–WM interactions during overtly performed complex grasping movements. We appreciate and encourage future research to replicate the current study, improve the limitations and extend the current findings.

In conclusion, the current study provides the initial neurophysiological investigation of the movement re-planning–WM interactions during grasping movements. It is shown that movement re-planning interferes with the verbal and visuospatial domains and entails re-planning costs. The current re-planning costs are operationalized by the reduced memory performance for the verbal and visuospatial tasks as well as longer movement execution times and larger P300 for the re-planned movements during the maintenance process. The current study extends the previous behavioral findings by highlighting the functional importance of the maintenance process for the manual action flexibility–WM interactions. Moreover, it extends the previous ERP findings by highlighting the distinct neurophysiological interactions of the movement re-planning with WM (compared to the movement execution). Moreover, the current study shows that P300 is generated not only during the re-planning of simple movements such as keypress but also during the re-planning of complex grasping movements. More generally, the current study contributes to a better understanding of the neurocognitive mechanisms underlying manual action flexibility.

## Supplementary Information

Below is the link to the electronic supplementary material.

Supplementary file1 (DOCX 27 KB)

## Data Availability

The datasets obtained and analyzed during the current study are available from the corresponding author on a reasonable request.

## Footnotes

1 For other P300-related perspectives such as surprise and prediction errors, see Donchin, 1981; Soh, & Wessel, 2020; Wessel, 2018 (for surprise) and Nassar, Bruckner, & Frank, 2019 (for prediction errors).

2 We also conducted the same statistical tests for the behavioral and EEG data before the outlier exclusion. Those statistical tests revealed similar results. Therefore, we reported and discussed the results based on the outlier exclusion in the article. In Appendix B, we reported the results before the outlier exclusion.

3 We conducted two additional statistical analyses concerning the maintenance process. First, we conducted a 2 × 2 × 5 repeated-measures ANOVA (*WM Task* × *Movement Planning* × *Electrode*) with a time window of 300–600 ms. This ANOVA revealed the results compatible with the original ANOVA with the time window of 200–700 ms. That is, there was a significant main effect of *Movement Planning (F* (1, 25) = 10.49*, p* = .003*, ω*^2^ = 0.27), in which ERP amplitudes were more positive in the re-planned condition (*M* = − 0.34, SD = 4.97) than the prepared condition (*M* = − 2.02, SD = 4.79) at all electrodes independent of the WM task (95% CI [0.61, 2.75]). These findings support the claim that our original findings were not bound to the specific time window chosen for the P300 analysis. Second, we conducted correlation analyses to examine any potential relationship between behavioral memory performance and P300 during the maintenance process. We averaged the performances for the verbal and visuospatial tasks across the prepared and re-planned conditions for the memory performance. Then, we subtracted the mean memory performance in the prepared condition from the mean memory performance in the re-planned condition. We obtained a memory performance difference between two movement conditions for each participant. Similar to the memory performance, we averaged the P300 amplitudes in the verbal and visuospatial tasks across the prepared and re-planned conditions at each electrode. Then, we subtracted the P300 amplitude in the prepared condition from the mean P300 amplitude in the re-planned condition. We obtained a P300 amplitude difference between two movement conditions at each electrode for each participant. We conducted correlation analyses on the obtained differences. Correlation analyses did not show any significant results at any electrode. We consider that the significant correlation between the memory performance and P300 based on the prepared and re-planned conditions would be interesting. At the same time, considering the complexity of the brain, we also consider that such a correlation would still be tentative. Nonetheless, future research should investigate such a correlation more profound to link the behavioral results with the ERP results, further enhancing the understanding of the neurophysiological correlates of the movement re-planning–WM interactions.
