# Supplementary material for: Manual action re-planning interferes with the maintenance process of working memory: an ERP investigation
Source: Psychol Res. 2022 Nov 24;87(6):1784–805. doi: 10.1007/s00426-022-01741-4 (PMC10366281; doi:10.1007/s00426-022-01741-4)
Supplement: Supplementary file 1 — Supplementary file1 (DOCX 27 KB) [file 426_2022_1741_MOESM1_ESM.docx]

**Appendix A**

The following tables show the data for each participant who was included in the grand-averaged ERPs. Trials Entered Epochs columns show the number of the trials entered in the epochs of the maintenance and retrieval processes after the initial exclusion of the trials (following the behavioral data analyses). Epochs Entered ERPs columns show the number of the epochs entered in the ERPs after the artefact detection and channel interpolation. Accordingly, these columns represent the final number of the epochs averaged for the individual ERPs for each experimental condition during the maintenance and retrieval processes.

**Table 1**

*Maintenance Process*

| Participants | Verbal Task | | | | | | | | Visuospatial Task | | | | | | | | |
| --- | --- | --- | --- | --- | --- | --- | --- | --- | --- | --- | --- | --- | --- | --- | --- | --- | --- |
|  | Trials Entered Epochs | | | | Epochs Entered ERPs | | | | Trials Entered Epochs | | | | Epochs Entered ERPs | | | | |
|  | Prepared | Re-planned | | Prepared | | Re-planned | | Prepared | | Re-planned | | Prepared | | Re-planned | | |  |
| 1 | 64 | | 30 | | 60 | | 30 | | 57 | | 29 | | 57 | | 29 | | |
| 2 | 69 | | 27 | | 69 | | 27 | | 66 | | 27 | | 65 | | 27 | | |
| 3 | 69 | | 29 | | 64 | | 25 | | 65 | | 29 | | 60 | | 29 | | |
| 4 | 69 | | 29 | | 63 | | 24 | | 68 | | 27 | | 66 | | 25 | | |
| 5 | 67 | | 28 | | 66 | | 25 | | 64 | | 29 | | 63 | | 26 | | |
| 6 | 67 | | 29 | | 65 | | 28 | | 68 | | 29 | | 63 | | 28 | | |
| 7 | 68 | | 27 | | 68 | | 26 | | 63 | | 28 | | 62 | | 28 | | |
| 8 | 64 | | 29 | | 64 | | 28 | | 67 | | 28 | | 62 | | 28 | | |
| 9 | 65 | | 28 | | 56 | | 25 | | 68 | | 29 | | 64 | | 29 | | |
| 10 | 66 | | 29 | | 66 | | 29 | | 68 | | 29 | | 64 | | 29 | | |
| 11 | 58 | | 28 | | 58 | | 28 | | 63 | | 27 | | 63 | | 27 | | |
| 13 | 69 | | 29 | | 68 | | 28 | | 43 | | 19 | | 43 | | 19 | | |
| 14 | 68 | | 29 | | 65 | | 28 | | 66 | | 29 | | 62 | | 26 | | |
| 15 | 65 | | 28 | | 65 | | 28 | | 65 | | 29 | | 68 | | 23 | | |
| 16 | 69 | | 28 | | 62 | | 23 | | 69 | | 28 | | 68 | | 23 | | |
| 17 | 67 | | 26 | | 50 | | 24 | | 68 | | 28 | | 65 | | 28 | | |
| 18 | 67 | | 27 | | 66 | | 27 | | 68 | | 25 | | 65 | | 28 | | |
| 21 | 66 | | 25 | | 66 | | 25 | | 67 | | 29 | | 66 | | 25 | | |
| 22 | 67 | | 29 | | 67 | | 29 | | 68 | | 29 | | 68 | | 29 | | |
| 23 | 69 | | 27 | | 64 | | 23 | | 67 | | 28 | | 57 | | 23 | | |
| 24 | 69 | | 29 | | 67 | | 29 | | 67 | | 29 | | 67 | | 29 | | |
| 25 | 68 | | 28 | | 46 | | 22 | | 67 | | 28 | | 51 | | 21 | | |
| 26 | 69 | | 29 | | 69 | | 29 | | 64 | | 29 | | 63 | | 28 | | |
| 27 | 68 | | 29 | | 66 | | 29 | | 66 | | 29 | | 59 | | 27 | | |
| 28 | 68 | | 29 | | 68 | | 29 | | 69 | | 30 | | 65 | | 28 | | |
| 29 | 69 | | 29 | | 69 | | 29 | | 66 | | 28 | | 66 | | 28 | | |
| 30 | 67 | | 30 | | 56 | | 26 | | 69 | | 28 | | 68 | | 28 | | |
| 31 | 69 | | 29 | | 66 | | 26 | | 65 | | 28 | | 65 | | 28 | | |
| 32 | 68 | | 29 | | 63 | | 29 | | 69 | | 26 | | 69 | | 25 | | |
| 33 | 67 | | 17 | | 48 | | 16 | | 67 | | 28 | | 63 | | 25 |  |  |

*Note:* Following participants were excluded from the statistical analyses: 15, 16, 17, 21 from 2x2x2x2 repeated measures ANOVA; 15, 16, 17, 27 from 2x2x5 repeated measures ANOVA.

**Table 2**

*Retrieval Process*

| Participants | Verbal Task | | | | | | | Visuospatial Task | | | | | | |  |
| --- | --- | --- | --- | --- | --- | --- | --- | --- | --- | --- | --- | --- | --- | --- | --- |
|  | Trials Entered Epochs | | | Epochs Entered ERPs | | | | Trials Entered Epochs | | | Epochs Entered ERPs | | | |  |
|  | Prepared | | Re-planned | Prepared | | Re-planned | | Prepared | Re-planned | | Prepared | Re-planned | | | |
| 1 | | 64 | 30 | | 63 | | 28 | 57 | | 29 | 55 | | 29 |  |  |
| 2 | | 69 | 27 | | 67 | | 27 | 66 | | 27 | 66 | | 27 |  |  |
| 3 | | 69 | 29 | | 59 | | 24 | 65 | | 29 | 61 | | 26 |  |  |
| 4 | | 69 | 29 | | 60 | | 24 | 68 | | 27 | 64 | | 24 |  |  |
| 5 | | 67 | 28 | | 67 | | 27 | 64 | | 29 | 62 | | 26 |  |  |
| 6 | | 67 | 29 | | 64 | | 28 | 68 | | 29 | 65 | | 28 |  |  |
| 7 | | 68 | 27 | | 67 | | 27 | 63 | | 28 | 63 | | 28 |  |  |
| 8 | | 68 | 27 | | 67 | | 27 | 67 | | 28 | 63 | | 27 |  |  |
| 10 | | 66 | 29 | | 66 | | 28 | 68 | | 29 | 67 | | 28 |  |  |
| 11 | | 58 | 28 | | 55 | | 27 | 63 | | 27 | 62 | | 27 |  |  |
| 13 | | 69 | 29 | | 69 | | 28 | 43 | | 19 | 43 | | 19 |  |  |
| 14 | | 68 | 29 | | 54 | | 22 | 66 | | 29 | 56 | | 20 |  |  |
| 15 | | 65 | 28 | | 65 | | 28 | 65 | | 29 | 48 | | 21 |  |  |
| 16 | | 69 | 28 | | 50 | | 21 | 69 | | 28 | 60 | | 19 |  |  |
| 18 | | 67 | 27 | | 62 | | 27 | 68 | | 25 | 65 | | 23 |  |  |
| 19 | | 68 | 28 | | 56 | | 23 | 64 | | 30 | 57 | | 26 |  |  |
| 20 | | 68 | 29 | | 48 | | 20 | 68 | | 30 | 62 | | 27 |  |  |
| 21 | | 66 | 25 | | 65 | | 24 | 67 | | 29 | 62 | | 28 |  |  |
| 22 | | 67 | 29 | | 64 | | 28 | 68 | | 29 | 68 | | 28 |  |  |
| 24 | | 69 | 29 | | 65 | | 28 | 67 | | 29 | 66 | | 29 |  |  |
| 26 | | 69 | 29 | | 63 | | 29 | 64 | | 29 | 61 | | 28 |  |  |
| 28 | | 68 | 29 | | 68 | | 29 | 69 | | 30 | 63 | | 29 |  |  |
| 29 | | 69 | 29 | | 65 | | 28 | 66 | | 28 | 63 | | 26 |  |  |
| 30 | | 67 | 30 | | 54 | | 24 | 69 | | 28 | 67 | | 28 |  |  |
| 31 | | 69 | 29 | | 68 | | 27 | 65 | | 28 | 65 | | 28 |  |  |
| 32 | | 68 | 29 | | 66 | | 27 | 69 | | 26 | 69 | | 26 |  |  |
| 33 | | 67 | 17 | | 53 | | 15 | 67 | | 28 | 63 | | 27 |  |  |
| 34 | | 69 | 28 | | 64 | | 24 | 64 | | 29 | 64 | | 29 |  |  |

*Note:* Following participants were excluded from the statistical analyses: 16, 19 from 2x2x2x2 repeated measures ANOVA; 16, 19, 21 from 2x2x5 repeated measures ANOVA.

**Appendix B**

The following table shows the results of the statistical tests conducted before the outlier exclusion. These tests reveal the significant results as reported in the Result section (based on the outlier exclusion). Therefore, the table does not include the retrieval process analyses. Moreover, it does not include the movement execution time analyses (no outlier exclusion).

**Table 1**

*Results of the Statistical Tests before the Outlier Exclusion*

|  | df | F | *p* | ω^2^ |
| --- | --- | --- | --- | --- |
| **Memory Performance Analysis** |  |  |  |  |
| 2x2 ANOVA |  |  |  |  |
| *WM Task* | 35 | 92.29 | .000 | .72 |
| *Movement Planning* | 35 | 17.18 | .000 | .28 |
|  |  |  |  |  |
| **Maintenance Process Analyses** |  |  |  |  |
| 2x2x2x2 ANOVA |  |  |  |  |
| *Movement Planning* | 29 | 7.94 | .009 | .19 |
| 2x2x5 ANOVA |  |  |  |  |
| *Movement Planning* | 29 | 15.08 | .001 | .32 |

*Note:* Thirty participants were entered in the grand-averaged ERPs for the maintenance process based on the 30% criterion.
